# Hot Spot Mutation in TP53 (R248Q) Causes Oncogenic Gain-of-Function Phenotypes in a Breast Cancer Cell Line Derived from an African American patient

**DOI:** 10.3390/ijerph13010022

**Published:** 2015-12-22

**Authors:** Nataly Shtraizent, Hiroshi Matsui, Alla Polotskaia, Jill Bargonetti

**Affiliations:** 1 Department of Biological Sciences, Hunter College, City University of New York, New York, NY 10065, USA; nataly.shtraizent@mssm.edu (N.S.); polotskaia@genectr.hunter.cuny.edu (A.P.); 2 Icahn School of Medicine at Mount Sinai, New York, NY 10029, USA; 3 Department of Chemistry, Hunter College, City University of New York, New York, NY 10065, USA; hmatsui@hunter.cuny.edu

**Keywords:** TNBC, African American, p53, impedance, deformability

## Abstract

African American (AA) breast cancer patients often have triple negative breast cancer (TNBC) that contains mutations in the *TP53* gene. The point mutations at amino acid residues R273 and R248 both result in oncogenic gain-of-function (GOF) phenotypes. Expression of mutant p53 (mtp53) R273H associates with increased cell elasticity, survival under serum deprivation conditions, and increased Poly (ADP ribose) polymerase 1 (PARP1) on the chromatin in the AA-derived TNBC breast cancer cell line MDA-MB-468. We hypothesized that GOF mtp53 R248Q expression could stimulate a similar phenotype in the AA-derived TNBC cell line HCC70. To test this hypothesis we depleted the R248Q protein in the HCC70 cell line using shRNA-mediated knockdown. Using impedance-based real-time analysis we correlated the expression of mtp53 R248Q with increased cell deformability. We also documented that depletion of mtp53 R248Q increased PARP1 in the cytoplasm and decreased PARP1 on the chromatin. We conclude that in the AA-derived TNBC HCC70 cells mtp53 R248Q expression results in a causative tumor associated phenotype. This study supports using the biological markers of high expression of mtp53 R273H or R248Q as additional diagnostics for TNBC resistant subtypes often found in the AA community. Each mtp53 protein must be considered separately and this work adds R248Q to the increasing list of p53 mutations that can be used for diagnostics and drug targeting. Here we report that when R248Q mtp53 proteins are expressed in TNBC, then targeting the gain-of-function pathways may improve treatment efficacy.

## 1. Introduction

It is important to study the biology of triple negative breast cancer (TNBC) because it is a difficult-to-treat disease due to a lack of targetable biomarkers [1]. Additionally TNBC is a health disparity in the African American population and is currently without a specific treatment [2]. The tumor suppressor protein p53 (also termed tumor protein 53, TP53) is mutated in more than 50 percent of human tumors [3]. In aggressive triple-negative sub-types of breast cancer the frequency of p53 mutations is 80 percent [4,5]. The genetic variations in *p53* can result in variable p53 isoforms that have the potential to influence the phenotype of the breast cancer [6]. The p53 protein can be (1) wild-type; (2) loss-of-function mutant; (3) non-expressed due to a deletion; or (4) oncogenic gain-of-function (GOF) mutant. These GOF mtp53 proteins result from “hot spot” missense mutations that occur in many cancers [7]. When the mutant p53 is oncogenic GOF, there is the possibility of being able to target the stable protein for inactivation, as well as blocking the activated signal transduction pathways. Therefore determining the hot spot GOF mtp53 proteins, expressed in TNBCs derived from African American patients, that drive GOF phenotypes through specific pathways paves the way to improved diagnostic and treatment paradigms. As early as 1991 mtp53 was suggested as a potential biological marker for breast cancer [8], but to date oncogenic mtp53 is not used as a breast cancer diagnostic or a target for breast cancer treatment.

There are a number of different GOF mutations found in the *p53* gene that promote tumorigenesis [6]. Two notable hot spot mutant p53 residues that associate with GOF in cancer are R273 and R248. We recently reported a simple method for measuring cell deformability and reported increased deformability mediated by mtp53 R273H in an AA-derived breast cancer cell line (MDA-MB-468) [9]. This deformability detection method implements triggering cells to expand upon hyposmotic shock and recording the change in volume by an impedimetric microsensor [9,10]. The more deformable cells are, the greater the change in impedance during cell swelling, and this corresponds to increased migratory and invasive potential [11,12]. This deformability also correlates with the fact that mtp53 R273H in breast cancer promotes increased transcription of cholesterol biosynthesis genes [13], which can potentially affect fluidity of the plasma membrane. Moreover we recently documented through a proteomics screen that mtp53 in TNBC increases cholesterol biosynthesis enzymes and increases poly (ADP ribose) polymerase 1 (PARP1) on the chromatin [14]. This increased PARP1 on the chromatin associates with increased sensitivity to PARP inhibitors [14]. Coupling mtp53-based detection methods with targeted therapeutic possibilities has the potential to improve TNBC outcomes.

It is important to determine if AA breast cancers that express other hot spot GOF mutant p53 proteins have similar associated increased deformability as well as other mtp53 associated phenotypes. The AA-derived breast cancer cell line HCC70 expresses the mtp53 R248Q. How mtp53 R248Q impacts breast cancers has not been determined. When R248Q and R248W were compared for GOF properties by expression in the non-small cell lung cancer cell line H1299, which has no endogenous p53, only R248Q promoted increased cell migration [15]. The R248Q mutation also promotes accelerated tumor onset and shorter lifespan in a humanized mouse model [16]. Therefore we predicted R248Q would also promote increased flexibility and the association of PARP with the chromatin.

In normal cells p53 serves as a guardian of genomic stability [17]; in cancer cells expression of mtp53 is associated with decreased stringency of the DNA-damage checkpoint and accumulation of genomic mutations [18,19]. Some mutations in p53 can lead to a simple lack of wild-type transcriptional and tumor-suppressive activity, while others can lead to a gain of function (GOF) that actively promotes tumor growth [6]. The cancer genome atlas (TCGA) analysis of samples from breast cancer patients confirmed a strong association of aggressive phenotype breast cancer with an 80% incidence of TP53 mutations [4]. However, further studies are required to precisely identify the mutation specific association and to advance the development of personalized therapy. In the current study we characterized the phenotype associated with mtp53 R248Q in breast cancer cells (HCC70) derived from an African American patient. Our study emphasizes the clinical potential of mtp53 detection and targeting for improved diagnostics and therapy of hard-to-treat cases of breast cancer. Herein we add mtp53 R248Q expression in TNBC to the factors that promote mtp53-associated breast cancer phenotypes.

## 2. Experimental Section

### 2.1. Cell Lines

MDA-MB-231, MDA-MB-468, MCF-7, HCC70 and HCC1806 were purchased from ATCC (Manassas, VA, USA; cat# HTB-26, HTB-132, HTB-22, CRL2315, CRL-2335, respectively). 10(1) and 3(4) mouse embryo fibroblasts were used and are as previously described [20].

### 2.2. Generation of shRNA-p53 Knockdown

All cells were grown in DMEM medium (Invitrogen, Carlsbad, CA, USA), supplemented with 10% FBS (Gemini, West Sacramento, CA, USA) and 2500 units of penicillin-streptomycin (Mediatech, Herndon, VA, USA) at 5% CO_2_ 37 °C humidified incubator. We generated constructs with inducible (doxycyclin-ON) shRNA for p53 (STGM-shp53) or control vector (STGM) as previously described [21]. The STGM.p53 shRNA (2120) targeting construct was designed with a doxycycline inducible green fluorescent protein gene followed by an endogenous mir30 shRNA with (or without) the incorporated p53 shRNA sequence to target the 3′UTR. Therefore GFP expression indicates mir30 expression but only indicates mtp53 knockdown for the STGM.p53 shRNA (2120). The constructs were introduced into the cells by a retrovirus mediated gene transfer method. Briefly, Phoenix packaging cells were transfected by the calcium phosphate method with an rtTA plasmid, the STGM-shp53 plasmid or STGM vectors. The generated viruses were harvested and cells were co-infected with virus containing rtTA plasmid and one of the vectors. After selection with puromycin (STGM vector) and hygromycin (rtTA), clonal cell lines were generated by a limited dilution method. Clonal cell lines were selected based on the level of p53 knockdown.

### 2.3. Protein Expression Analysis—Whole Cell Extracts

Cells were lysed in RIPA buffer (0.1% SDS, 1% NP-40, 0.5% deoxycholate, 150 mM NaCl, 1 mM EDTA, 0.5 mM EGTA, 50 mM Tris-Cl pH8) with 1 mM PMSF, 8.5 μg/mL aprotinin and 2 μg/mL leupeptin. A total of 50 μg of protein extract were separated by 10% SDS-PAGE and electro-transferred to nitrocellulose membrane. Immunoblotting was carried out with p53 antibodies (a 1:1:1 mix of hybridoma supernatants, pAb421, pAb240 and pAb1801), mouse anti-PARP (BD Biosciences, Franklin Lakes, NJ), fibrillarin (Abcam, Cambridge, MA), and the loading control was assessed using either anti-Actin antibody (Sigma, Cream Ridge, NJ; A2066) or anti-GAPDH (Santa Cruz, Dallas, TX; sc-25778).

### 2.4. Chromatin Fractionation

Cells were harvested and fractionation was performed using the Stillman protocol [22]. Briefly, after removing the media, cells were rinsed with cold PBS twice, scraped from the plates, pelleted by centrifugation in 50 mL tubes at 1000 rpm 5 min. Cell pellets were suspended in buffer A (10 mM HEPES pH 7.9, 10 mM KCl, 1.5 mM MgCl_2_, 0.34 M sucrose, 10% glycerol, 1 mM DTT, 0.5 mM PMSF, 2 μg/mL leupeptin, 8.5 μg/mL aprotinin) with 0.1% Triton X-100. After 5 min incubation on ice cells were spun down at 3600 rpm for 5 min at 4 °C. The supernatant was spun down for an additional 5 min at 13,000 rpm at 4 °C to clarify (cytoplasmic fraction). Pellets were washed 2 times with Buffer A by centrifugation at 3600 rpm for 5 min at 4 °C. Resuspention of the nuclear pellet was in Buffer B (3 mM EDTA, 0.2 mM EGTA, 0.5 mM PMSF, 2 μg/mL leupeptin, 8.5 μg/mL aprotinin) and then incubated on ice for 30 min with vigorous vortexing every 5 min and spun down at 4000 rpm for 5 min at 4 °C. The supernatant was nuclear soluble proteins and was maintained as a fraction and the pellet, enriched in chromatin, was washed 2 times with Buffer B, resuspended in buffer B and sonicated 3 times for 30 s followed by 30 s rest on ice (chromatin fraction). Samples were stored at −80 °C prior to loading on a gel.

### 2.5. Impedimetric Measurements/Deformability Measurements

For the analysis of deformability we used a commercial impedance analyzer, xCELLigence. 16-well plate (e-plate 16, ACEA Biosciences, Inc., San Diego, CA, USA) was incubated with 100 μL of poly-l-lysine for 30 min, rinsed with deionized water and dried in a cell culture hood under UV light. On a poly-l-lysine coated plate in quadruplicate, 2000 cells were plated per well. Following 2 h of cell stabilization on plates at 37 °C, culture media was replaced with dH_2_O and the impedance measurement was recorded every 2 s for 10 min. Impedance values were automatically converted to Cell Index (CI), relative impedance change at every measurement point. The xCELLigence system measures impedance at three discrete frequencies, 10 kHz, 25 kHz, and 50 kHz and it plots averaged impedance CI values among these frequencies.

### 2.6. Immunofluorescence

Cells were seeded onto glass cover slips at 10% confluency and after indicated treatments, washed once with 1× PBS and fixed with 4% paraformaldehyde in 1× PBS for 15 min at room temperature. Fixed cells were permeabilized with 0.5% Triton-X-100 in 1× PBS/1% FBS for 10 min at room temperature and washed three times with 1× PBS/1% FBS. The cells were incubated for 1 h at room temperature with 1:200 dilution of primary antibody or rabbit anti-human pre-immune serum, p53 mouse anti-human monoclonal DO-1. The slides were washed three times with 1XPBS/1% FBS, incubated with 1:400 dilution of Alexa-conjugated secondary antibody (Invitrogen) 1 h at room temperature and washed again three times with 1XPBS/1% FBS. Coverslips were then mounted onto slides using Vectashield mounting medium containing 4′,6-diamidino-2-phenylindole (DAPI) to visualize the nuclei.

### 2.7. Spinning Disk Confocal Microscopy

Cells were visualized using spinning disk confocal microscope. All confocal images were captured with the 60× objective. Images were visualized and captured under different channels: rhodamine (red), FITC, and ultra violet light (blue for DAPI).

### 2.8. Cell Culture in Matrigel

Cells were seeded at a density of 5 × 10^3^ cells per chamber in an eight chamber slide on top of 50 μL solidified matrigel (BD Biosciences) in MEBM basal medium without phenol red (Lonza CC-3153, Walkersville, MD, USA) supplemented with bullet kit components except for BPE (Lonza CC-4156), 10% charcoal FBS and 2% matrigel, in the presence of 10 nM estrogen and in the absence or presence of 2 μg/mL doxycycline. Medium was changed every three days.

### 2.9. Colony Size Quantification

Bright field/GFP images were taken using Nikon inverted microscope and area of the colonies was measured using ImageJ software. The number of pixels representing colony size was divided by 100 and grouped into <100, 100–500, 500–1000 and >1000. The number of colonies in each group was compared.

### 2.10. Statistical Analysis

We used Anova to compare cell impedance prior and following mtp53 knockdown. We used *t*-test for all comparison of cell proliferation and survival. *P*-value <0.05 was considered statistically significant.

## 3. Results and Discussion

### 3.1. Mutant p53 R248Q is Elevated in HCC70 Cells Similarly to Other Variants of mtp53

**Figure 1:**
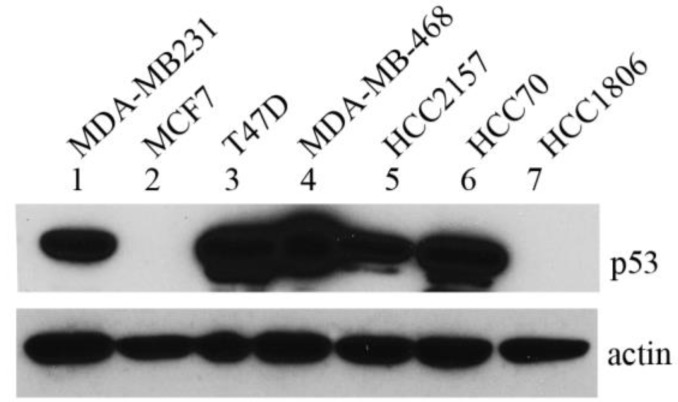
mtp53 is highly expressed in breast cancer cell lines. Expression of p53 protein in a panel of breast cancer cell lines: MDA-MB-231, MCF7, T47D, MDA-MB-468, HCC2157, HCC70 and HCC1806 and (lanes 1–7, respectively). 50 μg of protein extracted from different cell lines using RIPA buffer, were separated by gradient 4%–12% SDS/PAGE and analyzed by western blot using antibody to p53. Actin was used as a loading control. A representative blot is shown.

We compared the mtp53 protein expression levels in a number of established human breast cancer cell lines (see Table 1 for details). The p53 expression panel included a comparison of wt as well as R248Q, R248W, R273H, null, R280K and C194T mutants. Stable mtp53 protein expression was detected by western blot analysis of whole cell extracts from the human breast cancer cell lines MDA-MB-231, T47D, MDA-MB-468, HCC2157, and HCC70 (Figure 1, lane 1 and 3–6 respectively). In the HCC1806 cell line mtp53 has a 2bp insertion, causing no p53 protein to be detected (Figure 1, lane 7). In the MCF-7 cell line the p53 expressed is wild-type and therefore was present at a low level that was not detectable at the exposure shown (Figure 1, lane 2). In a collaborative study we previously showed that R273H and R280K confer gain-of-function characteristics to two TNBCs breast cancer cell lines, MDA-MD-468 and MDA-MB-231 respectively [13] We were interested to determine if the AA-derived breast cancer cell lines HCC70 with R248Q and HCC1806 with no p53 expression showed increased deformability when compared to the wild-type p53 expression cell line MCF-7.

**Table 1:** Panel of breast cancer cell lines. Types of human breast cancer cell lines used for comparison of p53 protein expression levels. TP53 mutation status and cell line characteristics are listed.

Cell Line	P53 Status	Charcteristivs
HCC70	R248Q	AA, TNBC
HCC2175	R248W	AA, TNBC
MDA-MB-46	273	AA, TNBC
HCC1809	Null (2bp insertion)	AA, TNBC
MD-MB-231	R280K	Non-AA, TNBC
T47D	C194T	Non-AA
MCF7	wt	Non-AA

### 3.2. The HCC70 Cell Line Shows Increased Deformability

**Figure 2:**
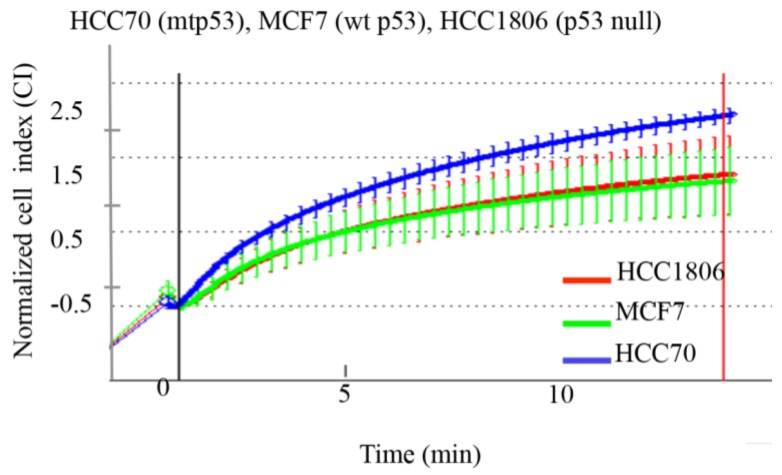
Expression of mtp53 in the HCC70 cell line is associated with increase cell deformability compared to cell lines with wt or no p53. Average variations of the impedance (CI) as a function of time and resulting swelling are shown. Mtp53-expressing cell lines (HCC70, red), wt p53-expressing cell line (MCF7, green) and no p53-expressing cell line (HCC1806, blue) were measured under hypotonic stress using the xCELLigence system. Cell volume (as Normalized Cell Index) was recorded every 2 s following induction of swelling, as described in materials and methods. The HCC70 cell line (red) showed significantly higher deformability, compared to MCF7 and HCC1806 cell lines (green and blue, respectively). Average values and standard deviation at each time points are shown (*n* = 4). The first vertical line indicates the addition of water. The second vertical line (shown in red) was used to generate slopes of the curves (data not shown).

We are able to detect the GOF mtp53 phenotype of R273H and R280K in MDA-MB-468 cells and MDA-MB-231 cells by impedimetric detection under hyposmotic pressure [9]. We therefore asked if the expression of mtp53 R248Q in the AA-derived HCC70 cell line demonstrated increased deformability when compared to the p53 null AA-derived breast cancer cell line HCC1806 and the wild-type p53 expressing cell line MCF-7. We observed increased deformability of HCC70 cells (blue line), while the MCF-7 and HCC1806 cells showed lower and very similar deformability kinetics (Figure 2).

### 3.3. Knockdown of mtp53 R248Q in HCC70 Cells

**Figure 3:**
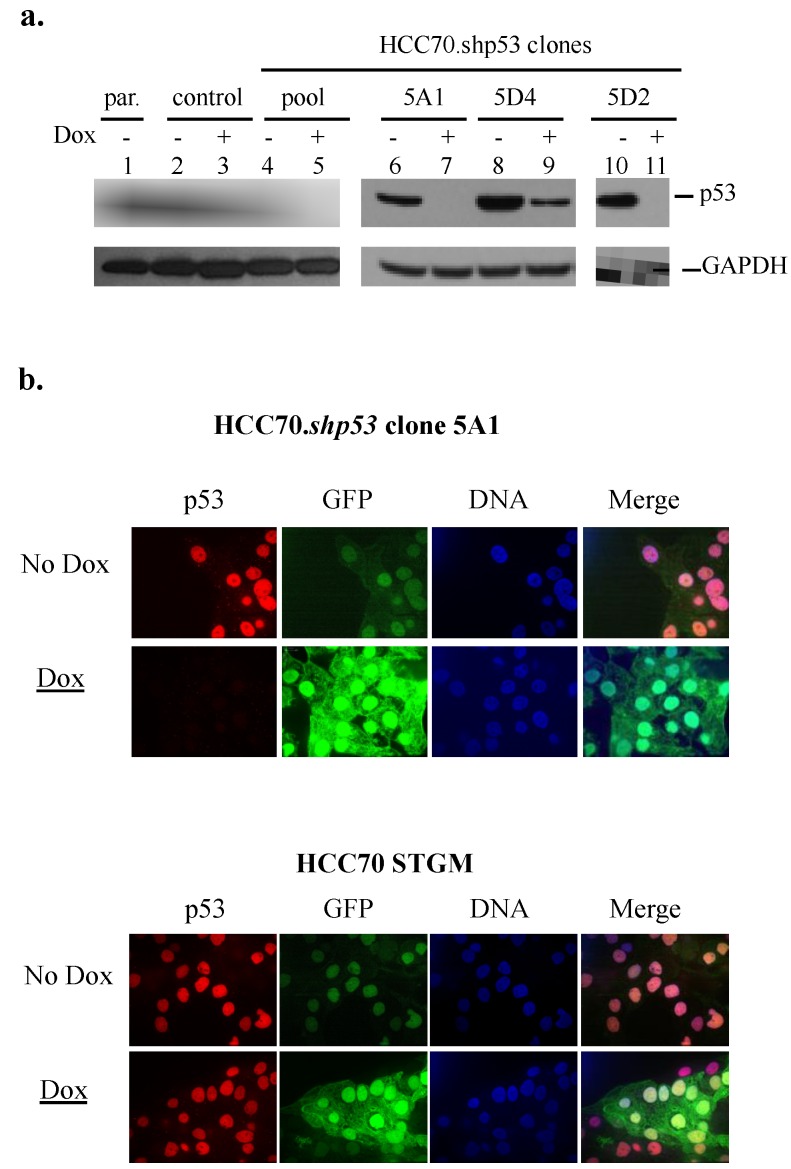
Inducible depletion of mtp53 proteins R248Q in breast cancer cell line HCC70. (**a**) The mtp53 expression in whole-cell extracts from parental HCC70 cell line (lane 1) and cell lines expressing vector control (control, lane 2,3) or mtp53-targeted shRNA (lanes 4–11), including HCC70. shp53 italic or not—Must be consistent pool (lane 4,5) and clonal cell lines (HCC70.shp53 5A1, lanes 6,7; HCC70.shp53 5D4, lanes 8,9 and HCC70. shp53 5D2, lanes 10,11) is shown. Cells were grown in the presence or absence of 8 μg/mL doxycycline (Dox as indicated) for 6 days. Whole-cell lysates were prepared as described in the Materials and Methods section, and 50 μg of protein was separated by gradient 4%–12% SDS/PAGE and analyzed by western blot using antibody to p53. GAPDH was used as a loading control. Expression of mtp53 in parental and control cell line are also shown (lanes 1 and 2 respectively); (**b**) confocal microscopy images of mtp53 protein were obtained by using anti-p53 antibody. DAPI staining was used to determine the nucleus, and GFP was an indicator of doxycycline-mediated induction. Nuclear immunofluorescence representing mtp53 in the HCC70.shp53 5A1 cell line reduced following doxycycline (top panel) and did not change in the control cell line (bottom panel).

In order to further examine the associated actions of the mtp53 R248Q in the HCC70 cells we used our established protocol to genetically engineer mir30-based shRNA knockdown derivatives of HCC70 (from now on called HCC70.shp53) [21]. We used shRNA mediated knockdown to test if the expression of mtp53 R248Q in the HCC70 cell line was directly responsible for the increased deformability. First we analyzed the efficiency of the knockdown in the HCC70.shp53 pool and next in three clonal cell lines (Figure 3a,b). The expression of mtp53 was significantly decreased by the addition of doxycycline in the HCC70.shp53 pool, as well as clones 5A1, 5D4 and 5D2 (Figure 3a, compare lanes 4 to 5, 6 to 7, and 10 to 11). No change in mtp53 level was detected following doxycycline addition to the HCC70.STGM vector control cell line (Figure 3a, compare lane 2 to 3). The clone 5A1 showed an excellent knockdown of mtp53 R248Q. This knockdown was validated further by immunofluorescence imaging which showed that following doxycycline treatment the HCC.shp53 clone 5A1 had increased expression of GFP and reduced mtp53 staining (Figure 3b). HCC70 STGM control cells showed no doxycycline-mediated decrease in mtp53 staining (Figure 3b).

**Figure 4:**
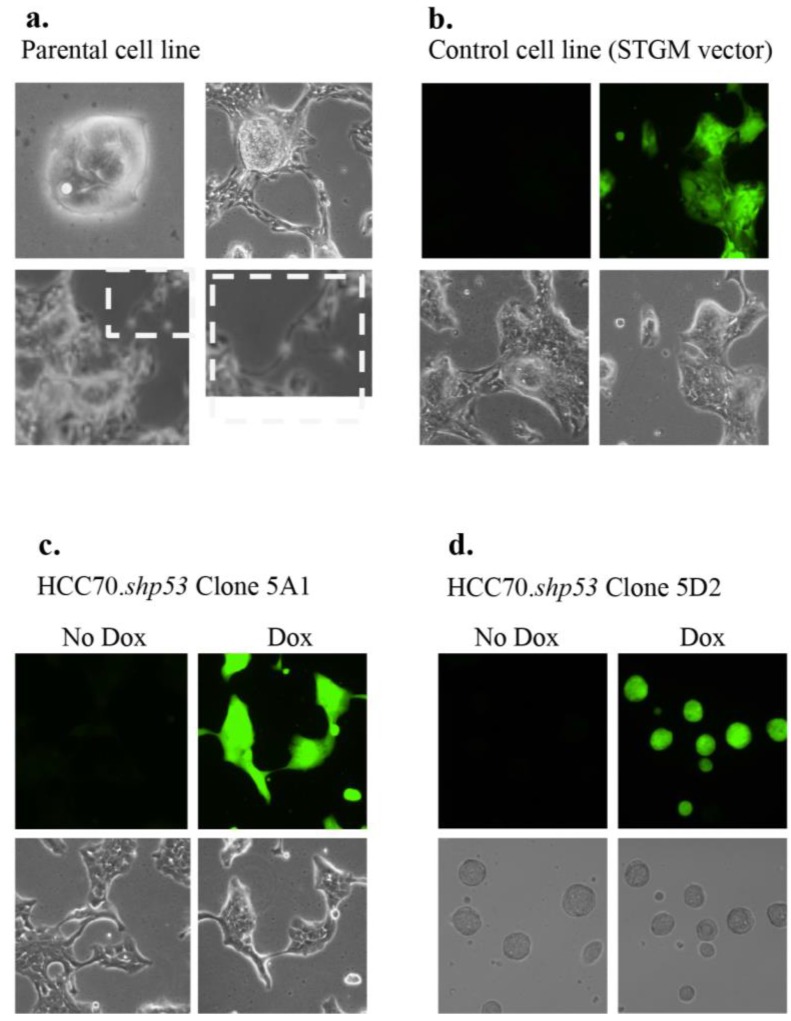
Morphology of HCC70.shp53 5A1 and 5D2 diverge from that of parental cell line. (**a**) Images of light microscopy of live HCC70 parental cell (boxed panel shows increased magnification on the right of the same region boxed on the left) and (**b**) STGM-control cell lines are shown. Fluorescent microscopy showing GFP indicates that Dox was added to the culture conditions. In the STGM-control there was no mtp53 knockdown. During cloning of the HCC70.shp53 pool we isolated clones that were representative of each type of morphology. (**c**) Specifically, HCC70.shp53 5A1 exhibited branched colony structures only (bottom left) and (**d**) HCC70.shp53 5D2—stem cell-like round colony morphology.

The HCC70 cell line morphology was difficult to characterize and depended on the density of the cells (Figure 4a). When sparsely plated, the HCC70 parental cell line exhibited cells densely packed together with no distinguishable borders between them. Single cells shed off of the colonies and once a higher density was achieved the outer layer of cells in each colony branched out to contact the neighboring colony, while the central part of the colony continued to form a densely packed group of cells (Figure 4a). The cell morphology in cells infected with STGM control vector was the same as the parental cell line with or without Dox (Figure 4b). During establishing of clonal cell lines from HCC70. shp53*.* pool culture we isolated two morphologically distinct clones (5A1 and 5D2), which collectively represented the mixed morphology of the parental cell line (Figure 4c,d). The knockdown of mtp53 did not influence the cell morphology.

### 3.4. Deformability of the HCC70 Cell Line Depends on Expression of mtp53 R248Q and on the Morphology of the Cells

**Figure 5:**
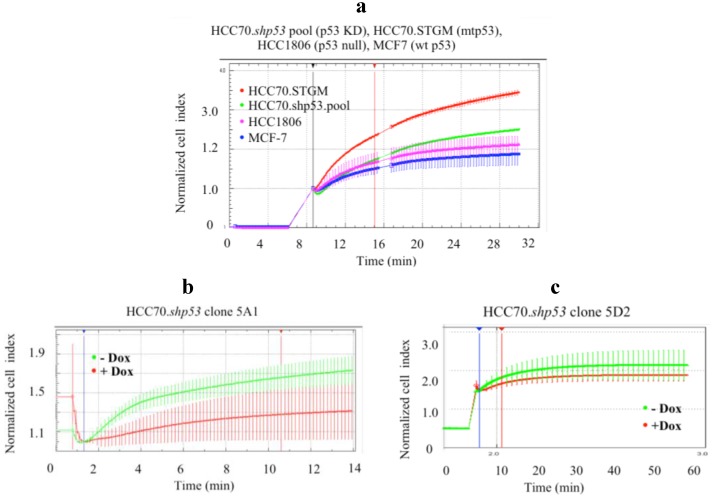
Cell deformability decreased upon mtp53 KD in pool population and in clonal cell lines with branched mesenchymal-like morphology. (**a**) Deformability (Normalized Cell Index) of HCC70 control cell line (red) or HCC70.shp53 pool (green), treated with dox, was compared to MCF7 (blue) and HCC1806 (fuchsia) cell lines, as described in the Materials and Methods section. Following mtp53 knockdown in HCC70.shp53 pool cell line change in CI during swelling was significantly reduced compared to the HCC70 control cell line. (**b**,**c**) Average variations in deformability of clonal cell lines HCC70.shp53 5A1 (**b**) and 5D2 (**c**) were compared prior and following mtp53 knockdown (green and red, respectively). Mtp53 knockdown in (**b**) HCC70.shp53 clone 5A1 significantly decreased the ability of cells to swell (**c**) while in the clone 5D2 very little impedance change was detected. Average values and standard deviation at each time points are shown (*n* = 4). The first vertical line indicates the addition of water. The second vertical line (shown in red) was used to generate slopes of the curves (data not shown).

The clonal cell lines were assessed for impedance changes during hyposmotic pressure to see if HCC70.shp53 recapitulated the reduction of mtp53 resulting in increased stiffness [9]. In the pool population of HCC70.shp53 we observed decreased deformability when compared to the STGM control cell line (Figure 5a). Prior to mtp53 knockdown the HCC70.shp53 cells were less deformable that either MCF-7 or HCC1806 (Figure 5a). The knockdown of R248Q in HCC70.shp53 clone 5A1 decreased cell deformability (Figure 5b). However the knockdown of R248Q mtp53 in clone 5D2 did not detectably influence cell deformability (Figure 5c). Therefore clonal differences acting together with p53 knockdown caused variability in the outcomes.

### 3.5. Temperature Sensitive mtp53-Val135 Confers Increased Cell Flexibility

**Figure 6:**
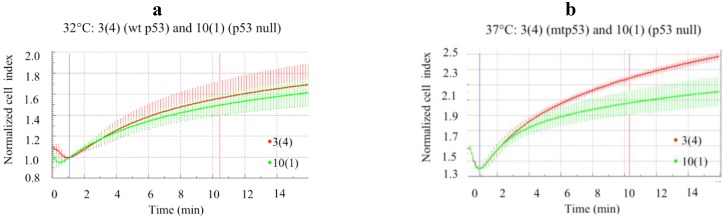
Increase in expression of GOF mtp53 was associated with increased deformability. Change in cell impedance (expressed as normalized cell index) in isogenic p53 null cell line 10(1) or in the cell line 3(4) expressing ts p53 is shown. Cell lines were cultured at 32 °C for wtp53 conformation or at 37 °C to induce the mtp53 conformation in the 3(4) cell line, as described elsewhere [20]. (**a**) The normalized cell index (*i.e.*, change in impedance) of 10(1) (green) and 3(4) (red) cells cultured at 32 °C were recorded to assess the effect of wt p53 expression on cell deformability and no differences were observed; (**b**) Impedance variations in 10(1) (green) and 3(4) (red) cells cultured at 37 °C (green) were recorded to assess the effect of mtp53 conformation expression on cell deformability. Average values and standard deviation at each time points are shown (*n* = 4). The first vertical line indicates the addition of water. The second vertical line (shown in red) was used to generate slopes of the curves (data not shown).

To confirm that GOF mtp53 increases cell deformability we employed isogenic cell lines which express temperature sensitive mtp53 (called 3(4)) and compared them to the parental p53-null cell line (called 10(1)) [20]. Temperature sensitive mtp53-Val135 has a wild-type conformation at 32 °C and a GOF mutant confirmation at 37 °C [23]. When the parental line 10(1) was compared to the 3(4) cell line with the p53 in the wild-type conformation the cell deformability showed no difference (Figure 6a). However when the two cell lines were maintained at 37 °C so the mtp53-Val135 was expressed in the GOF conformation, we observed 3(4) cells with increased cell deformability (Figure 6b). This was evident by the 3(4) cells showing a higher normalized cell index over time (compare the red line to the green line in Figure 6b). The rationale for this experiment was to compare one additional mutant p53 isoform that is known to confer a gain-of-function that is temperature dependent. Here we see this that this directly correlates with increased impedance.

### 3.6. The Knockdown of mtp53 R248Q in HCC70 Changes Cell Morphology

**Figure 7:**
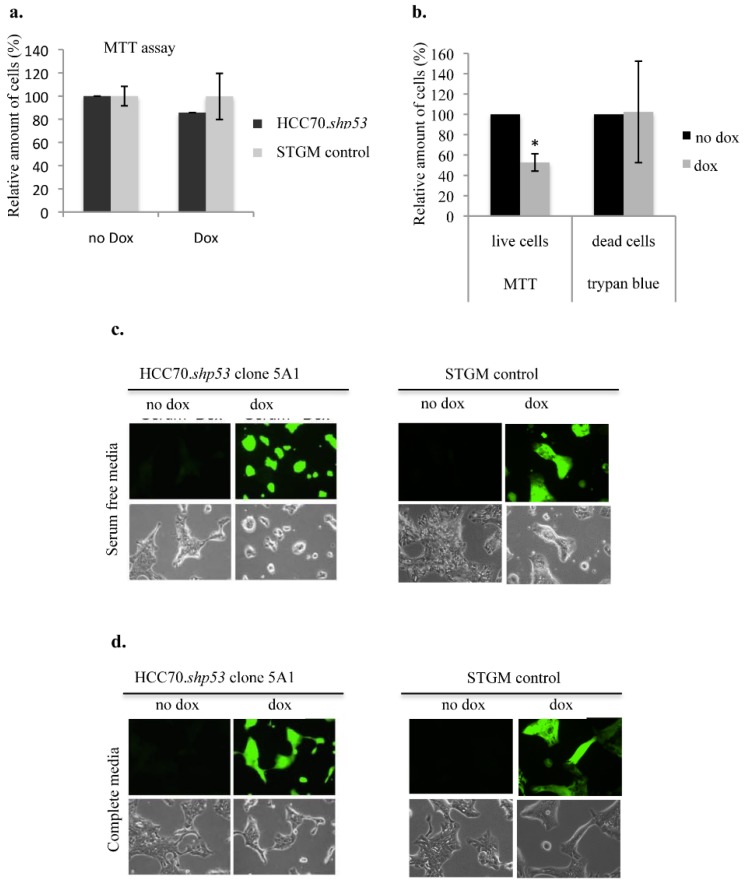
Loss of branched colony morphology following mtp53 knockdown during culture in serum free media. (**a**) MTT assay of the cell viability following dox treatment in HCC70.*shp53* and STGM-control cell lines (black and grey bars, respectively). (**b**,**c**) Cell morphology was assessed in serum deprivation conditions following mtp53 KD. (**b**) MTT assay and trypan blue in HCC70.*shp53* cells following serum starvation and with a comparison between the cells prior and following mtp53 knockdown (black and grey bars, receptively). (**c**) Images of light and fluorescent microscopy of HCC70.*shp53* clone 5A1 and STGM control treated with or without dox and cultures in serum deprived conditions. **(d**) Images of light and fluorescent microscopy of HCC70.*shp53* clone 5A1 and STGM control treated with or without dox and cultures in complete medium.

We examined the ability of mtp53 R248Q to confer a survival advantage on HCC70 under low serum stressful conditions (Figure 7). We first tested if knockdown of mtp53 R248Q (by doxycycline treatment) under normal cell culture conditions (cell culture media conditioned with 10% FBS) reduced cell viability in the HCC70.shp53 pool cell line. Doxycycline (Dox) treatment did not reduce the metabolic activity of the mitochondria as shown by MTT assay (Figure 7a). We then examined the influence of serum deprivation on clone 5A1 that showed a decrease in flexibility with mtp53 knockdown. Serum deprivation conditions for the HCC70.shp53 clone 5A1 with p53 knockdown decreased mitochondrial activity but did not influence the trypan blue staining (Figure 7b). This suggested that mtp53 knockdown decreased the metabolic activity of the cells but did not increase cell death. We observed that the morphology of serum starved HCC70.shp53 with p53 knockdown became more round compared to the non-treated cells (Figure 7c). There was no difference observed for the serum starved STGM control cell line treated with Dox (Figure 7c). Finally under normal serum conditions the treatment of HCC70.shp53 cells with Dox did not influence the cell morphology (Figure 7d). Therefore the lack of serum in the absence of mtp53 expression was a contributor for the loss of the mtp53-associated morphology phenotype and decreased the mitochondrial activity.

### 3.7. Knockdown of mtp53 in HCC70.shp53 5D2 Decrease Colony Size and PARP on the Chromatin

**Figure 8:**
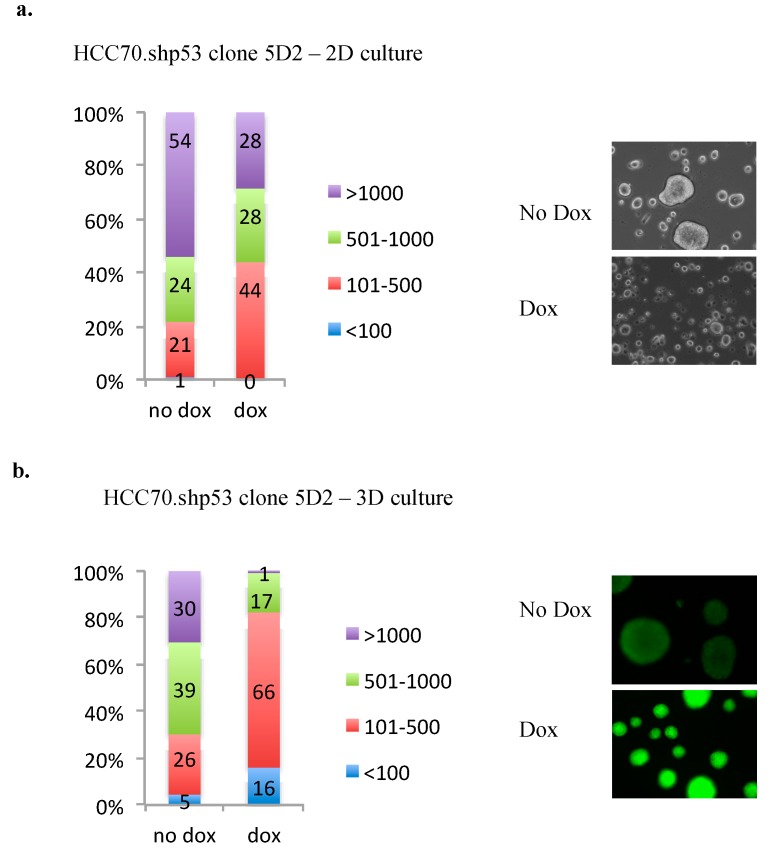
HCC70 colony size is reduced by the knockdown of R248Q mtp53. (**a**) Colony size of HCC70.*shp53* clone 5D2 treated with or without Dox, was quantified using ImageJ software and presented as a distribution of size grouped randomly into <100 (blue), 101–500 (red), 501–1000 (green) and >1000 (purple). 100 colonies were used in quantification and % colonies in each size group are presented in a bar graph. Representative light and fluorescent microscopy images are shown. (**b**) Colony size of HCC70.shp53 clone 5D2 treated with or without Dox and cultured on matrigel for 3D colony formation, was quantified using ImageJ software and presented as a distribution of size grouped randomly into <100 (blue), 101–500 (red), 501–1000 (green) and >1000 (purple). 100 colonies were used in quantification and % colonies in each size group are presented in a bar graph.

The HCC70.shp53 5A1 and 5D2 clones demonstrated some differences in flexibility and morphology. We were interested to determine if there were mtp53 associated phenotypes that could be detected in HCC70.shp53 5D2 that associated with mtp53 actions in a different clonal background. The 5D2 cells showed an interesting colony formation phenotype. We therefore asked if the 5D2 clone exhibited any of the other phenotypes previously associated with mtp53 R273H expression in MDA-MB-468 cells. MDA-468. shp53 cells show a mtp53-driven PARP association with the chromatin [14] and increased colony size when grown in 3D culture [13]. The knockdown of mtp53 in HCC70.shp53 5D2 reduced the colony size during cell culture in either 2-dimensions (2D) or 3-dimensions (3D) (Figure 8a,b). These colony formation assays were only carried out with 5D2 and not 5A1, in order to determine if there was a mtp53 associated phenotype in 5D2 cells.

**Figure 9:**
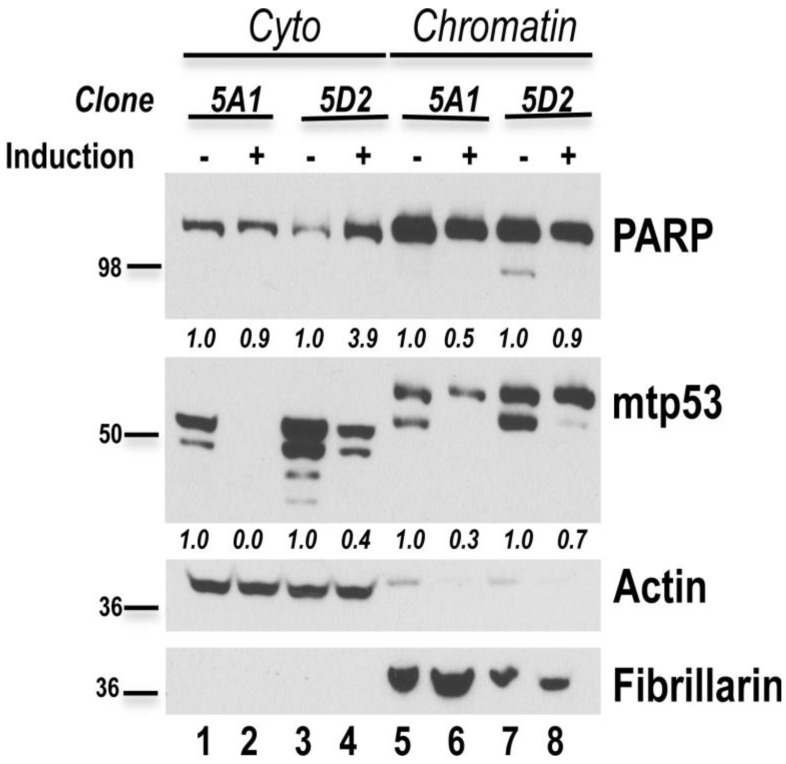
Depletion of mtp53 modulates PARP expression. HCC70.*shp53* clones 5A1 and 5D2 were grown in the presence or absence of 6 μg/mL of doxycycline for 7 days and fractionation was carried out. Samples were resolved on a gradient 4%–12% SDS/PAGE which resulted in the observation of differentially migrating p53 in cytoplasmic and chromatin fractions [14]. A total of 50 μg of protein from the cytoplasmic fraction and 10 μg of the chromatin fraction per lane were resolved. Protein levels of p53, PARP1, actin, and fibrillarin in the fractions were determined by western blot analysis and the results were quantified by ImageJ. Actin was used to normalize the cytoplasmic fractions and fibrillarin was used to normalize the chromatin fraction (relative expression values comparing with and without knockdown are shown under the PARP1 and mtp53 western blots).

In order to address a previously reported nuclear cytoplasmic associated mutant p53 gain-of-function property we addressed the level and localization of PARP [14]. We previously observed many different bands and variable mobility of mutant p53 in the cytoplasmic and chromatin fractions when they are analyzed on a gradient gel; these bands suggest different p53 post-translational modifications [14]. Furthermore, knockdown of R273H in MDA-MB-468 cells causes PARP to move from the nucleus to the cytoplasm [14]. Depletion of R248Q in the 5A1 and 5D2 cells caused PARP1 to be reduced on the chromatin (Figure 9, compare lane 5 to 6 and lane 7 and 8). Image J analysis demonstrated a greater reduction of chromatin associated PARP1 in 5A1 cells following mtp53 knockdown. Interestingly clone 5D2 showed a greater increase in cytoplasmic PARP1 when mtp53 was depleted, and this correlated with our past findings for R273H (compare lane 3 to 4). Therefore the mtp53 depletion phenotypes of increased cytoplasmic PARP and decreased flexibility are not directly related.

## 4. Conclusions

Herein we have shown that depletion of mutant p53 R248Q in the AA-derived cell line HCC70 decreased the elasticity of the breast cancer cells of a pool culture and one clonal cell line 5A1. We also observed decreased colony size of the HCC70.shp53 clone 5D2. For both 5A1 and 5D2 clones following mtp53 knock down we observed increased PARP in the cytoplasm and decreased the PARP on the chromatin. The expression of mtp53 R248Q and the associated increase in chromatin associated PARP and increased deformability can potentially serve as surrogate biomarkers of aggressive TNBC disease and thus can potentially be used to assess the effectiveness of mtp53 targeted therapy. The current study complements our previous observations that certain mtp53 proteins can promote gained oncogenic phenotypes to breast cancer cells [9,13,14]. These phenotypes include (but are not limited to) enhanced expression of cholesterol biosynthesis enzymes, increased PARP on the chromatin, and increased cell flexibility. The diagnostic and therapeutic potential of these properties can be coupled to genotyping specific breast cancers for their corresponding mtp53 status in order to deliver precision medicine.

It was interesting that we observed that the knockdown of mtp53 R248Q confered reduction in flexibility to the clone HCC70.shp53 clone 5A1 but not to 5D2 (Figure 5). The mtp53 knockdown was similar in both clones so this result had something to do with clonal differences in combination with mtp53 activities. When mtp53 was knocked down the 5D2 clone did show a change in two other mtp53 associated phenotypes. Following mtp53 knockdown the colony size of 5D2 was reduced (Figure 8). Moreover following p53 knockdown, both clones 5A1 and 5D2 displayed a decrease in nuclear PARP and an increase in the cytoplasmic PARP (Figure 9). While two different clones displayed variability in cell flexibility, it is important to note that the introduction of temperature sensitive mutant p53 in mouse cells increased cell flexibility. We saw that the temperature sensitive mtp53-Val135 increased the flexibility of MEFS when the p53 protein was expressed in the mutant conformation (Figure 6). We do not know the mechanism that causes the two clones of HCC70. shp53, 5A1 and 5D2, to not display the same phenotype of decreased flexibility following p53 knockdown. We speculate that it may have something to do with the cell morphology of the human cells the two clones were derived from. The different cell morphology at the beginning of selection may indicate signaling already in place prior to the p53 knockdown. All the data we have collected is presented here and it includes HCC70.shp53 pools as well as different clones. While the reason for variability of different clones is not yet determined the combination of mtp53-associated outcomes examined herein: (a) the change in flexibility; (b) cell metabolism under serum deprivation conditions; (c) colony sizes; and (d) the PARP localization following R248Q knockdown provides data indicating that the R248Q point mutation of p53 is a GOF allele in TNBC. This conclusion adds one additional mtp53 to the list of potential TNBC diagnostic and targeting tools.

In our proteomics screen with MDA-MB-468 cells we found that Paxillin is amongst numerous proteins that sustain level changes associated with the expression of mtp53 R273H [14]. While it is possible that increased cholesterol biosynthesis enzyme production increases cell flexibility it is also possible that Paxillin plays a role in changes in flexibility [14]. A recent study by Sero *et al*. is an example of how expression of Paxillin can modify morphology of cells [24]. Changes in cell shape are associated with changes in cell properties, such as proliferation, survival and metastatic potential [24,25] and therefore cell morphology is one of the criteria for pathology analysis and cancer prognosis. We suggest that cell flexibility assessment and p53 genotyping can be used to better diagnose aggressive TNBCs. The advantage of targeting mtp53 R273H and R248Q GOF properties in breast cancers may be that it will allow specific targeting of TNBCs with less damage to normal cells. Many aggressive TNBCs are found in AA women. While the breast cancer cell lines MDA-MB-468 cells and HCC70 cells are only two examples of AA-derived TNBCs, the fact that they express two different hot spot p53 mutations that associate with strong GOF phenotypes allows speculation that more AA-TNBCs will be driven by mtp53. The high association of mtp53 with TNBC is a fact that should become an actionable and targetable breast cancer biomarker for people of all races [4]. By targeting the genomics of breast cancers without regard to self-identified race we will do a better job at determining the drivers, and best targets, of all breast cancers. Detecting and targeting mtp53 driven pathways will be one small step at better reducing TNBC health disparities.
